# Chronic Exercise Increases Plasma Brain-Derived Neurotrophic Factor Levels, Pancreatic Islet Size, and Insulin Tolerance in a TrkB-Dependent Manner

**DOI:** 10.1371/journal.pone.0115177

**Published:** 2014-12-22

**Authors:** Alberto Jiménez-Maldonado, Elena Roces de Álvarez-Buylla, Sergio Montero, Valery Melnikov, Elena Castro-Rodríguez, Armando Gamboa-Domínguez, Alejandrina Rodríguez-Hernández, Mónica Lemus, Jesús Muñiz Murguía

**Affiliations:** 1 Centro Universitario de Investigaciones Biomédicas, Universidad de Colima, Colima, México; 2 Facultad de Medicina, Universidad de Colima, Colima, México; 3 Depto de Patología, Instituto Nacional de Nutrición y Ciencias Médicas "Salvador Zubirán,” México City, México D.F.; Tohoku University, Japan

## Abstract

**Background:**

Physical exercise improves glucose metabolism and insulin sensitivity. Brain-derived neurotrophic factor (BDNF) enhances insulin activity in diabetic rodents. Because physical exercise modifies BDNF production, this study aimed to investigate the effects of chronic exercise on plasma BDNF levels and the possible effects on insulin tolerance modification in healthy rats.

**Methods:**

Wistar rats were divided into five groups: control (sedentary, C); moderate- intensity training (MIT); MIT plus K252A TrkB blocker (MITK); high-intensity training (HIT); and HIT plus K252a (HITK). Training comprised 8 weeks of treadmill running. Plasma BDNF levels (ELISA assay), glucose tolerance, insulin tolerance, and immunohistochemistry for insulin and the pancreatic islet area were evaluated in all groups. In addition, *Bdnf* mRNA expression in the skeletal muscle was measured.

**Principal Findings:**

Chronic treadmill exercise significantly increased plasma BDNF levels and insulin tolerance, and both effects were attenuated by TrkB blocking. In the MIT and HIT groups, a significant TrkB-dependent pancreatic islet enlargement was observed. MIT rats exhibited increased liver glycogen levels following insulin administration in a TrkB-independent manner.

**Conclusions/Significance:**

Chronic physical exercise exerted remarkable effects on insulin regulation by inducing significant increases in the pancreatic islet size and insulin sensitivity in a TrkB-dependent manner. A threshold for the induction of BNDF in response to physical exercise exists in certain muscle groups. To the best of our knowledge, these are the first results to reveal a role for TrkB in the chronic exercise-mediated insulin regulation in healthy rats.

## Introduction

Exercise is a powerful method of improving insulin sensitivity and metabolic-related diseases [1], [2] because it enhances the pancreatic islet insulin content in diabetic rodent models [3]. In rodents, acute exercise (swimming or running) increases insulin sensitivity in the skeletal muscle [1], [4], [5], [6] and liver [7], and running decreases phosphoenolpyruvate carboxykinase expression in the liver [8]. Moreover, the levels of Akt and Foxo1 phosphorylation (a signaling pathway and insulin-regulated transcription factor, respectively) significantly increase after acute treadmill running [9].

Brain-derived neurotrophic factor (BDNF), a member of the nerve-growth factor-related family of neurotrophins [10], [11], [12], promotes neuronal development and function in the peripheral and central nervous systems [13]. In the peripheral nervous system, neurotrophins also play a key role in the regulation of neuronal survival [14]. The effects of neurotrophins are mediated by a family of specific transmembrane tyrosine kinase receptors, of which, TrkB is the primary signal transduction receptor for BDNF [15], [16]. Acute and chronic physical exercise increase BDNF expression in central (spinal cord) and peripheral tissues (skeletal muscle) in rodents [17]–[19], [20]. In addition to the effects of BDNF on the nervous system, this neurotrophin reportedly exerts direct effects on the pancreas, liver, white adipose tissue, and skeletal muscles [21], [22], [23], [24], all of which are important organs in glucose homeostasis.

In diabetic mice, subcutaneous BDNF enhances insulin activity in the liver [22]. The insulin receptor (IR) phosphorylation levels and phosphatidylinositol–3 kinase (PI3K) activity in the skeletal muscle were observed to increase after the peripheral administration of BDNF [22]. Furthermore, BDNF elevates the insulin levels and restores granulated β cells in the pancreatic islets in diabetic mice [21], besides, it also modulates glucagon secretion from α cells [23]. Therefore, BDNF is tightly linked to glucose regulation.

Acute and chronic physical exercise augment BDNF production in rodents and humans [25], [26], [27]. In addition, increased BDNF levels were observed to correlate with enhanced exercise performance [28]. During acute exercise, the skeletal muscle produces and releases BDNF into the bloodstream [29], [30].The increases in insulin tolerance and plasma BDNF levels after chronic exercise suggest an important interaction between BDNF, glucose, and insulin regulation. In the present study, we analyzed the effects of a chronic 8-week treadmill running program on glucose metabolism and plasma BDNF levels. Furthermore, we studied how BDNF participated in chronic exercise-induced insulin tolerance.

## Materials and Methods

### Ethics statement

The experiments were performed in accordance with the United States National Institutes of Health Guideline for the Care and Use of Laboratory Animals. Animal studies and experimental procedures were approved by the Bioethics and Biosecurity Committee of the Faculty of Medicine and CUIB of the University of Colima (No 2012-05).

### Animals and training schedule

Male Wistar rats (2 months old; weight, 200–250 g) were individually housed in polyethylene cages in an environmentally controlled room (22°C–24°C) with a 12-h light/dark cycle. The rats were allowed free access to water and food (Teklad Global Diet: protein/fat/fiber, 18.0%/5.0%/5.0%). In some experiments, food was withdrawn 12 h before the experimental or surgical procedures. Surgical procedures were performed under intraperitoneal (i.p.) administration of sodium pentobarbital (3.3 mg/100 g in saline solution; Aranda, México). Animals were pre-sedated with buprenorphine (0.03 mg/kg body weight via subcutaneous injection; Temgesic, Schering-Plough, México) 5 min before the surgical procedures. The training schedule followed was that by Wisloff et al. [31], with some modifications. In brief, before the exercise program, all animals underwent a preconditioning running regimen for a week comprising 30 min of daily running at a speed of 15 m/min on a rat treadmill (Modular Treadmill Simplex, Mod. 42528; Columbus Instruments, Columbus, Ohio) installed in a ventilated acrylic cage (38×14×13 cm). An electrified grid (0.6 mA intensity) was placed behind the belt of the treadmill to induce running. Rats that failed to run regularly were excluded from the training protocol. The exercise program involved three sessions per week for an 8-week period. Every training session began with a 10-min warm-up (18 m/min), after which the speed was progressively increased to the respective speed (for 60 min, see protocol section) and ended with a 10-min recuperation period (18 m/min). Under these conditions, the training ensured 60%–80% of the maximal oxygen consumption. The entire experiment was completed in 8 weeks. The trained rats ran at a constant inclination angle of 25° because changes in this parameter would significantly affect the VO_2_ max [31]. Exercise sessions were always performed between 9 AM and 12 AM.

### Experimental protocol

**Figure 1:**
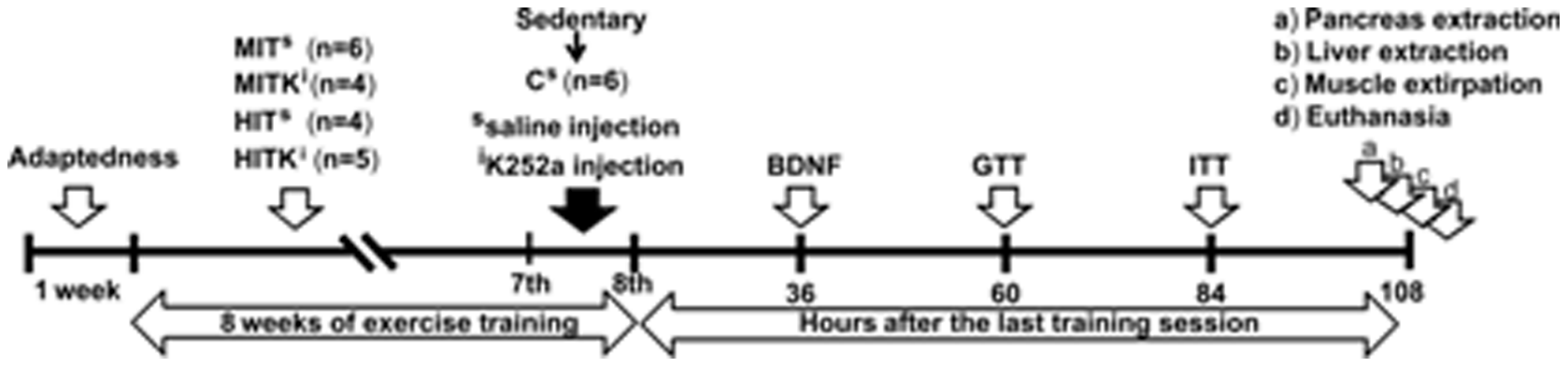
Experimental protocol. C, control rats; BDNF, brain derived neurotrophic factor; GTT, glucose tolerance test; ITT, insulin tolerance test; HIT, high-intensity trained rats; HITK, high-intensity trained rats that received i.p. injections of a BDNF receptor inhibitor K252a; MIT, moderate-intensity trained rats; MITK, moderate-intensity trained rats that received i.p. injections of K252a.

Twenty-five rats were randomly divided into the following groups: a) C group, included control rats that were kept sedentary but could freely move in their cages throughout the experiment (n = 6); b) MIT group, included rats subjected to moderate-intensity training (60% VO_2_ max) for 8 weeks (n = 6); c) HIT group, included rats subjected to high-intensity training (80% VO_2_ max; n = 4); d) MITK group, included rats subjected to exercise as in “b” along with daily i.p. injections of a TrkB inhibitor (K252a; 50 µg/kg body weight; Sigma-Aldrich México) during the seventh and eighth weeks of the experimental period (n = 4); and the e) HITK group, included rats subjected to exercise as in “c” along with daily i.p. injections of K252a as in “d” (n = 5). The C, MIT, and HIT groups received daily i.p. injections of saline instead of K252a during the seventh and eighth weeks of the experimental period. All groups were subjected to: intracardiac blood sampling to measure plasma BDNF concentration, a glucose tolerance test (GTT), an insulin tolerance test (ITT), the extraction of pancreatic tissue for immunohistochemistry, the extraction of liver tissue to measure glycogen after an insulin injection into the portal vein and the removal of muscle tissue to measure the *Bdnf* mRNA levels (Fig. 1). Upon extirpation of the gastrocnemius, soleus, and plantaris muscles, the rats were killed with an overdose of sodium pentobarbital.

### Measurement of plasma BDNF

Thirty-six hours (1.5 days) after the final training session and before surgical procedures, all rats were lightly anesthetized with sodium pentobarbital (2.5 mg/100 g body weight; i.p.) and 0.5 mL of venous blood were collected via cardiac puncture. Plasma BDNF concentrations were determined using an enzyme-linked immunosorbent assay (ELISA) kit (EK0308; Insight Genomics) according to the manufacturer's instructions. The samples were analyzed in duplicate and the mean concentrations were calculated. BDNF antibodies did not cross-react with any other cytokines. The limit of sensitivity was fixed at <2 pg/mL. Color was detected 30 min after the stop solution was added and the absorbance was read at 460 nm.

### Glucose tolerance test

The i.p. GTT was performed at 60 h (2.5 days) after the final training session and immediately after obtaining basal glucose samples from awake rats that had received i.p. injections of D-glucose (200 mg/100 g body weight) after a 12-h fasting period. The glucose concentrations were measured in tail vein blood samples using a glucometer (AccuChek Sensor Advantage; Roche, México) and test strips. Blood sampling was performed as follows: 1 sample was collected at the baseline (immediately before glucose injection at t = 0) and 4 more samples were collected at 15 min (0.25 h), 35 min (0.6 h), 95 min (1.6 h), and 135 min (2.25 h) after the glucose administration. Blood glucose response to GTT was calculated as the area under the glucose curve for each rat according to the trapezoidal method (GraphPad Prism FAQ82, USA).

### Insulin tolerance test

Eighty-four hours (3.5 days) after the final training session, awake, fed rats received i.p. injections of insulin lispro (0.08 U/100 body weight, Humalog, Lilly Mexico) immediately after collecting a basal glucose sample. Blood glucose concentrations were measured as in GTT at the baseline (immediately before insulin administration at t = 0 min) and at 30 min (0.5 h), 60 min (1 h), 120 min (2 h), and 180 min (3 h) postinjection. Blood glucose response to ITT was calculated as % basal, and as the area under the curve (AUC) in % for each rat according to the trapezoidal method; the data were normalized to C group.

### Immunohistochemistry and pancreatic islet analysis

To analyze the effects of physical exercise on the pancreatic islets, an immunohistochemical insulin analysis was used. In brief, 108 h (4.5 days) after the final training session, the pancreata from all studied groups were rapidly dissected to collect the duodenal region. This region was trimmed free of adherent fat and connective tissue and fixed overnight in a freshly prepared 10% buffered formaldehyde solution [32]. The tissue samples were ethanol-dehydrated and embedded in paraffin wax. Sections of 5 µm were cut on a rotary microtome and deparaffinized at 100°C for 20 min, transferred to xylene, ethano-xylene, and absolute alcohol, and finally washed in distilled water to be processed for immunohistochemical analysis [33]. The endogenous peroxidases and proteins were blocked using a Background Blocker (BioSB 0105, Sta. Barbara, CA, USA) for 5 min and rinsed for 2 min with TBS before a 45-min incubation with an antibody against insulin (100 µL of a 1∶200 dilution of the mouse monoclonal antibody HB125; Biogenex, San Ramon, CA, USA). The sections were then washed with TBS for 2 min; peroxidase activity was induced for 10 min with 2 drops of a mouse/rabbit immune detector biotin link (BioSB 0005, Sta. Barbara, CA, USA) after which the samples were rinsed in TBS for 2 min and incubated for 10 min with 2 drops of the mouse/rabbit immune detector HRPP LABEL (BioSB 0005). The brown colorimetric reaction was revealed with DAB and visualized under a microscope. The sections were subsequently washed and counterstained with hematoxylin for 30 s before mounting with Entellan (Merck, Darmstadt, Germany). The non-specific immunostaining was minimal when primary antiserum was omitted.

Labeled pancreatic sections were chosen at random and analyzed using an Axiocam MRC-5 model digital camera (Carl Zeiss, Germany) attached to an AxioPlan 2M model bright-field optical microscope (Carl Zeiss) with a motorized stage and A-plan 20× objectives (total magnification, 200x). Using the MosaiX and Autofocus modules, images of the entire sample surfaces were scanned and the islet areas were measured by outline spline. All images were obtained with the same lighting conditions and exposure times in AxioVs 40 V.4.7.0.0 imaging program (Carl Zeiss Imaging Solutions GmbH, 2006-200 imaging program, Germany).

### Glycogen determination

The presence of glycogen in the hepatocyte cytoplasm after insulin injection (performed immediately after collecting the pancreatic duodenal region) was detected and quantified using the periodic acid-Schiff staining (PAS staining kit, Merck, Germany) according to the manufacturer's instructions. The livers were removed as previously described [7] with some modifications. In brief, insulin (0.2 mL of 10^−6^M) was injected into the portal vein; after 10 min, a liver tissue fragment from the right lobule of the organ was collected and immediately placed in 10% buffered formaldehyde (freshly prepared) for 48 h, embedded in paraffin, sectioned at a 5-µm thickness, and assessed for glycogen within the hepatocytes using the PAS reaction. In this method, periodate oxidizes the hydroxyl moieties of glucose residues to aldehydes, which subsequently react with the Schiff reagent to generate a purple-magenta color. Six representative fields from at least 4 different liver fragments per rat (80 and 90 µm^2^) were analyzed under bright field optical microscopy (AxioPlan 2 M) and captured with a digital camera (Axiocam MRC-5; Carl Zeiss) attached to a motorized stage with a Planeofluor 40× objective. Images of the entire sample surfaces were scanned using the MozaiX and Autofocus modules in the Axiovision 4.8 software package for histomorphometric analyses and determinations of the glycogen-covered liver area. The glycogen signal was expressed as a percentage of the total tissue area. Background staining was calculated from diastase-treated slices.

### Muscle dissection

After the liver tissue collection, the gastrocnemius, soleus, and plantaris muscles from both posterior limbs were dissected and sequentially removed.

### BDNF expression levels in the muscle samples by real-time RT-PCR

After rapid thawing and weighing steps, the muscle samples (approximately 100 mg) were manually homogenized in 9 volumes of ice-cold physiological saline with a glass-Teflon homogenizer. The homogenates were centrifuged for 15 min at 3,000×*g* (5801R centrifuge; Germany) and the resulting supernatants were collected for mRNA expression analysis.

A total of 100 ng of total Trizol-isolated (Invitrogen, Carlsbad, CA) RNA from each sample were submitted to reverse transcription and polymerase chain reaction (RT-PCR) using a Lightcycler RNA master SYBR Green I kit (Roche, Nutley, NJ) and specific primers for the *Bdnf* gene or the constitutive gene beta-actin (*Actb*) [34].

The following primer sequences were used: *Bdnf*, sense: 5′-GAGCGTGTGTGACAGTATTAG-3′, antisense: 5′-GATTGGGTAGTTCGGCATTG-3′ and *Actb*, sense: 5′TGTCACCAACTGGGACGATA-3′ antisense: 5′GGGGTGTTGAAGGTCTCAAA-3′ Amplifications were performed using the LightCycler 1.5 system (Roche, Nutley, NJ). To determine the reaction efficiencies, standard curves for each gene were generated from several known amounts of RNA (0.6, 1.6, 8.0, 40, and 200 ng). The efficiency values were 1.8 for *Bdnf* and 1.7 for *Actb*. To characterize the amplification specificity, a melting curve analysis was performed for each reaction at the end of the 40^th^ cycle. This analysis indicated 1 product per gene. The threshold cycle (Ct) values were normalized to the values from the endogenous housekeeping gene *Actb* (ΔC_t_) and used to obtain the relative gene expression according to the formula 2^−ΔΔCt^, where ΔΔCt  =  [(Ct *_Bndf_* − Ct *_Actb_*)_ problem tissue_ − (Ct *_Bndf_* − Ct *_Actb_*) _control tissue_].

### Data presentation and statistical analysis

Numerical results are expressed as the means ± standard errors (S.E.) from the indicated numbers of experiments. All statistical analyses were performed using the statistical software package SPSS 17.0. Groups of data were compared in a one way analysis of variance (ANOVA) followed by Tukey's *post hoc* test; a *p* value of <0.05 indicated statistical significance. Statistical significance is shown in the figures as follows: *p<0.05 vs. the C group; **^#^** *p*<0.05 vs. the MIT group; **^§^** *p*<0.05 vs. the MITK group; and **^†^** *p*<0.05 vs. the HIT group.

## Results

### Plasma BDNF levels increased in response to chronic exercise

**Figure 2:**
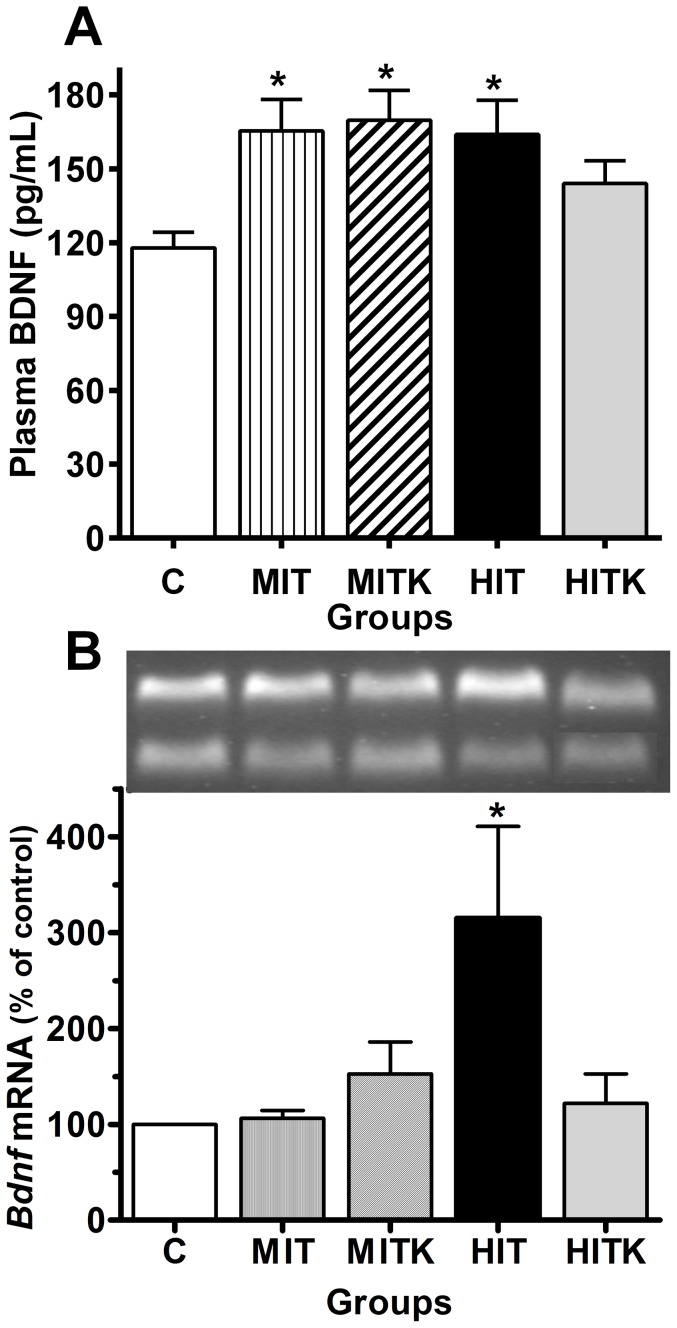
Effects of an 8-week treadmill exercise regimen on all studied groups shown in the protocol. BDNF levels in pg/mL (A) and relative BDNF expression in the soleus muscle (B). Real time RT-PCR reactions were performed with 100 ng of total RNA and specific primers. Representative PCR bands are shown in the gel enclosure; the larger band corresponds to the HIT group and the lower bands indicate the β-actin housekeeping gene expression. Values are shown as means ± S.E. from 6 independent samples; *p<0.05 vs. control, ANOVA and Tukey's tests.

Four groups of experimental rats were subjected to an 8-week running regimen and compared with sedentary controls (C). The MIT group was exposed to moderate-intensity training (22 m/min). The HIT group was exposed to high-intensity training (28 m/min). The MITK group was exposed to the MIT regimen with the addition of BDNF receptor inhibitor (K252a) injections during weeks 7 and 8. The HITK group was exposed to the HIT regimen with the addition of K252a injections during weeks 7 and 8. Thirty-six hours (1.5 days) after training, plasma BDNF concentrations were significantly higher in the trained groups than those in the sedentary controls; the mean BDNF concentration of the C group was 117.8±6.3 pg/mL, whereas those of the trained rats were 165.4±12.8 pg/mL in the MIT group, 169.7±12.1 pg/mL in the MITK group (*p*<0.05 for both groups vs. C), 164.0±13.8 pg/mL in the HIT group (*p*<0.05 vs. C), and 144.0±9.3 pg/mL in the HITK group (Fig. 2A) (*p*<0.05 vs. C). These results indicated that the circulating BDNF concentrations increased with chronic physical exercise.

### Bdnf mRNA expression increased in the muscles during exercise according to real-time RT-PCR

We observed that plasma BDNF levels increased in response to moderate and high-intensity exercise (Fig. 2A). Previous studies have suggested that muscle tissue is a source of BDNF during exercise [30]; therefore, we measured the *Bdnf* mRNA expression levels in the soleus muscle, a homogeneous slow-twitch muscle that is very active in running rats. This evaluation was performed 4.5 days after the final treadmill training session. A significant increase in *Bdnf* mRNA expression was observed in the HIT group (215±95.7%; *p*<0.01 vs. the C group) in the slow soleus skeletal muscle (Fig. 2B). No significant changes were detected in the plantaris and gastrocnemius muscles, although both showed a tendency toward increased expression (data not shown).

### Chronic exercise increased the glucose tolerance

To determine the glucose tolerance after chronic exercise, we injected glucose into all rats at 60 h (2.5 days) after the final treadmill session. The basal fasting arterial glucose levels (t = 0) were comparable in all studied groups: 4.2±0.1 mmol/mL in the C group, 3.8±0.1 mmol/L in the MIT group, 3.5±0.2 mmol/L in the MITK group, 4.1±0.1 mmol/L in the HIT group, and 3.8±0.1 mmol/L in the HITK group (*p* = 0.1)

**Figure 3:**
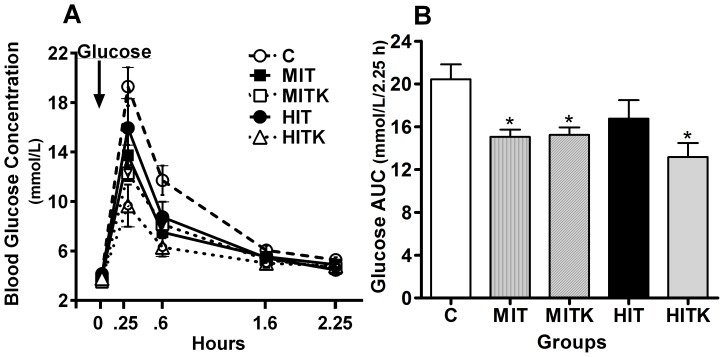
Glucose tolerance test in all experimental groups. All rats received i.p. injections of D-glucose (200 mg/100 g body weight) at the 0 time point. A) Glucose concentrations in mmol/L. B) Glucose concentrations from (A), expressed as area under the curve (AUC) in % basal during a 2.25-h period. Values are means ± S.E. from 6 independent samples; *p<0.05, ANOVA and Tukey's tests.

The glucose concentrations after i.p. glucose administration were consistently lower at all studied time points in all of the exercised groups relative to the controls (Fig. 3A). To better estimate the glucose tolerance, the area under the curve (AUC, Fig. 3B) of plasma glucose changes after glucose injection were calculated (20.5±1.4 mmol/L for the C group, 15.1±0.7 mmol/L for the MIT group [*p*<0.05 vs. the C group]; 15.3±0.7 mmol/L for the MITK group [*p*<0.05 vs. the C group]; 16.8±1.7 mmol/L for the HIT group, and 13.2±1.2 mmol/L for the HITK group [*p*<0.01 vs. the C group]; Fig. 3B). In all groups except HIT, chronic exercise significantly reduced plasma glucose levels, suggesting that rats exposed to running exhibited increased glucose tolerance.

### BDNF receptor activity was required for chronic exercise-induced insulin tolerance

**Figure 4:**
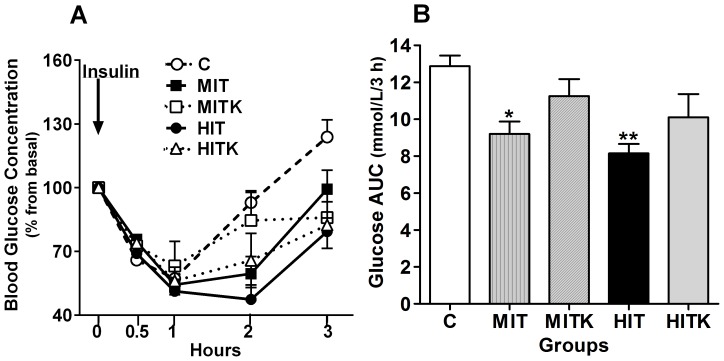
Insulin tolerance test in all experimental groups. All rats received i.p. injections of 0.08 mg insulin/100 g body weight at the 0 time point. A) Glucose concentrations expressed as % basal. B) Glucose concentrations from (A), expressed as the area under the curve (AUC) in % basal during a 3-h period. Values are shown as means ± S.E. from 6 independent samples; *p<0.05, ANOVA and Tukey' test.

Eighty-four hours (3.5 days) after running, the rats were injected with insulin, after which plasma glucose levels were measured at different time points (Fig. 4A). The basal fasting arterial glucose levels (t = 0) were comparable in all the studied groups: 5.1±0.2 mmol/L in the C group, 4.7±0.1 mmol/L in the MIT group, 4.5±0.1 mmol/L in the MITK group, 4.5±0.1 mmol/L in the HIT group, and 3.8±0.4 mmol/L in the HITK group, all these values represent a 100 percent. In all rats, insulin induced a decrease in the circulating glucose levels, that was most evident in the MIT and HIT groups, indicating higher insulin tolerance in these rats (Fig. 4A). To better integrate insulin tolerance over the duration of the experiment, we calculated the AUC (Fig. 4B). A significant decrease in the AUC was observed for the MIT and HIT groups. However, neither group that had been treated with K252a, the BDNF receptor inhibitor, during the final 2 weeks of training (MITK and HITK) significantly differed from the controls, as shown by the following values: the AUCs were 100% in the C group, 73.94±8.4% in the MIT group (*p*<0.05 *vs*. the C group), 90.08±9.9% in the MITK group (no significant differences *vs*. other groups), 63.67±9.9% in the HIT group (*p*<0.05 *vs*. the C group), and 80.32±11.59% in the HITK group (no significant differences *vs*. other groups) (Fig. 4B). These results indicate that chronic physical exercise enhanced insulin tolerance in a BDNF receptor activity-dependent manner.

### Pancreatic islet size increased after chronic physical exercise

**Figure 5:**
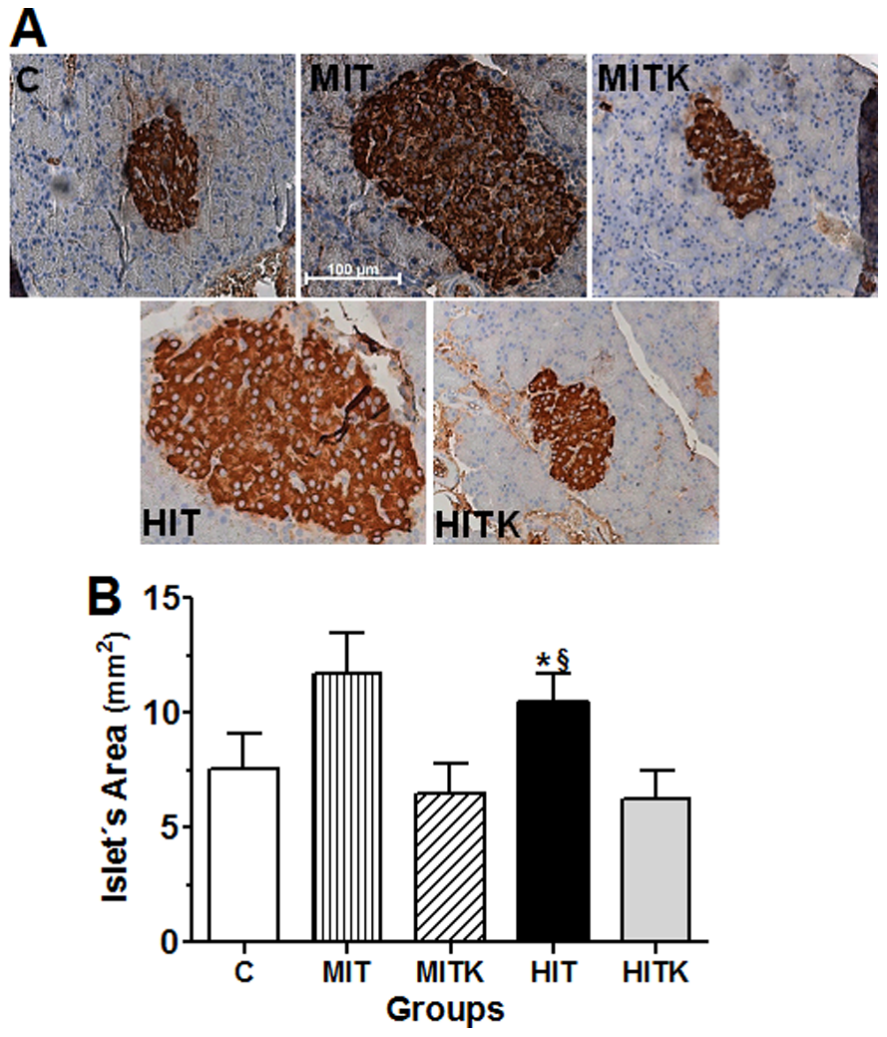
Pancreatic immunohistochemistry, effects of an 8-week treadmill exercise regimen in studied rats. (A) Insulin distribution and (B) pancreatic islet size. Light micrographs reveal the staining patterns of the pancreatic islets; the pancreatic islet areas (mm^2^) were evaluated in 209 islets. C, control sedentary group; CK sedentary rats that received K252a as in MITK and HITK groups; MIT, medium-intensity training rats; MITK, as MIT with a TrkB inhibitor (K252a) injection; HIT, high-intensity training rats; HITK, as HIT with a TrkB inhibitor injection; data are shown as means ± S.E. *p<0.05 *vs*. C; ^§^ *p*<0.05 *vs.* HITK; ANOVA and Tukey' tests.

Furthermore, rats were anesthetized 108 h (4.5 days) after the final treadmill session and a duodenal pancreatic fragments were excised and immunostained for insulin and islet size evaluations. Insulin staining clearly revealed round objects that corresponded to islets in the pancreata from all groups (Fig. 5A). The C and MIT groups showed similar insulin immunoreactivities, whereas the staining in the MITK group was slightly diminished, suggesting a requirement for BDNF receptor activity. Reduced insulin immunoreactivity was observed in the HIT and HITK groups (faintly stained cells), indicating that chronic high-intensity physical exercise reduced the pancreatic islet insulin contents. The average islet size was measured by random sampling from 5-µm thick paraffin sections because the immunoreactivity yielded a good contrast that demarcated the borders of individual Langerhans islets. In the C group, the average islet area was 7.5±1.5 mm^2^, whereas in the MIT and HIT groups, the islet areas were larger than those in the controls with respective values of 11.7±1.8 mm^2^ and 10.5±1.2 mm^2^. However, this difference was only significant in the HIT group (*p*<0.05). This effect was not observed when rats were under the effects of the TrkB inhibitor K252a. The islet areas in the MITK and HITK groups were 6.5±1.3 mm^2^ and 6.2±1.3 mm^2^, respectively, and these values did not significantly differ from the C group (Fig. 5B). To discard that TrkB inhibitor (K252a) affects by its own the beta cell mass, 3 new rats were added to the total, to do 3 control experiments in sedentary rats that received this inhibitor (CK rats), with a total of 59 islets evaluated. The average islet size in these rats was 6.74±0.94 mm^2^ (without significant difference *vs*. C group) (Fig. 5 A and B). These data indicate that chronic physical exercise induces an increase in the pancreatic islet size in an apparently BDNF receptor-dependent manner.

### Moderate exercise increases liver glycogen levels after portal insulin injection

**Figure 6:**
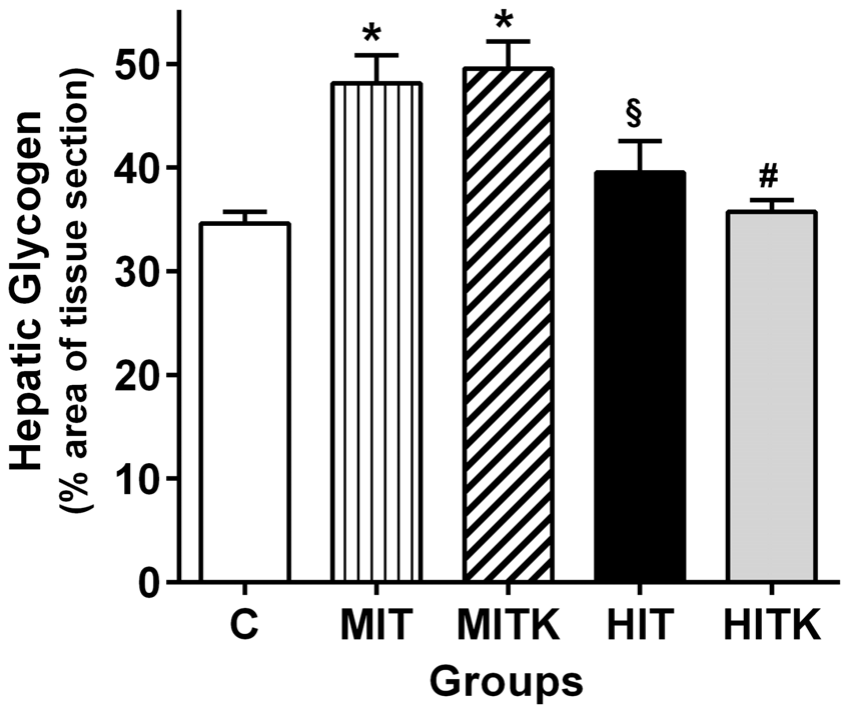
Hepatic glycogen levels in studied rats after insulin injection into the portal veins (0.2 mL 10^−6^M), effects of an 8-week treadmill exercise regimen. Liver fragments were stained according to the PAS method (n = 6 per group). Six representative fields from at least 4 different liver fragments per rat (80 and 90 µm^2^) were analyzed. Data are shown as means ± SEM. *p<0.05 *vs*. the C group, #*p*<0.05 *vs*. the MIT group, and §*p*<0.05 *vs*. the HITK group; ANOVA and Tukey's tests.

Prior to sacrifice (4.5 days after the final training session), the portal veins were cannulated in the anesthetized rats. Insulin was injected into the portal circulation system and after a 10-min interval, a fragment of the right liver lobe was excised for glycogen determination using PAS. A significant increase in the glycogen-occupied hepatic area was observed in both of the moderate-intensity exercise groups (MIT and MITK), with values of 34.6±1.2 in the C group and 48.1±2.7 and 49.6±2.6 for the MIT and MITK groups, respectively (*p*<0.001 vs. the C group). However, no significant changes in liver glycogen were observed following insulin administration in the high-intensity exercise groups regardless of TrkB inhibitor administration (HIT, 39.6±3.0; HITK, 35.7±1.2; *p*>0.05 vs. C; Fig. 6). The HIT and HITK livers had significantly lower glycogen occupation after insulin injection relative to the MIT and MITK livers, respectively (*p*<0.05) (Fig. 6). We conclude that moderate exercise resulted in increased insulin-induced liver glycogen storage in a BDNF-independent manner.

## Discussion

In this study we observed that an 8-week physical treadmill exercise regimen significantly increased plasma BDNF levels in an exercise intensity-independent manner. Insulin tolerance also increased after chronic physical exercise and was attenuated with TrkB receptor blockage. Chronic moderate and high-intensity exercise (MIT and HIT groups) induced pancreatic islet enlargement in a TrkB activity-dependent manner.

Thirty-six hours after completing the 8-week treadmill training program, the basal plasma BDNF levels increased near 40% in all the groups (Fig. 2A). The origin of the circulating BDNF following prolonged exercise, has not yet been clarified, although some of it is likely originate in the muscle as suggested by Sakuma and Yamaguchi [30]. However, in our experiments we only observed a significant increase in *Bdnf* mRNA in the HIT group (Fig. 2B). As the brain is the principal contributor to the circulating BDNF, and most of it derives from hippocampus region during exercise [26], it is probable that in our trained rats this neurotrophin is derived from the brain as well.

The rise in GTT observed in all exercised rats is consistent with previous reports using comparable protocols [35]. The physical exercise prevents hyperglycemia through multiple mechanisms, and the GTT primarily measures pancreatic insulin secretion in response to glucose [36], [37], [38]. In our trained rats the decrease in glycemia observed after a glucose challenge was not likely due to increased insulin secretion. Exercise training, similar to that used in this study, induces a reduction in pancreatic Gk mRNA expression without affecting GLUT 2 mRNA expression [39], furthermore, other studies in exercised rats show a reduced insulin secretion subsequent to glucose injections [40]. Physical exercise may increase GTT, augmenting muscle oxidative capacity, energy disposal, or blood flow [41], all of them important in muscle glucose uptake. In addition, exercise and muscular contraction activate AMP protein kinase (AMPK) [42], [43] to stimulate glucose uptake by skeletal muscle without affecting insulin levels [44]. Therefore, we suggest that in our trained groups, glucose tolerance did not occur at the pancreatic β-cells level, but at the muscular level and other glucose-dependent tissues.

Insulin tolerance was significantly greater in trained rats (MIT and HIT) (Fig. 4A and B), as previously reported [45], [46]. In this study, trained rats that received i.p. injections of K252a (MITK and HITK) exhibited a decreased insulin tolerance during the ITT, compared with rats without inhibitory treatment, probably due to a reduction of AMPK activity, regulated by BDNF in the muscle [29]. As AMPK activation plays a key role in free fatty acid (FFA) oxidation in the skeletal muscle [47], [48], TrkB receptor blockage may reduce the effect of BDNF in the MITK and HITK groups, likely caused by a decrease in FFA oxidation and AMPK activity [29]. Lower BDNF-AMPK activity in the skeletal muscle could induce increased diacylglycerol (DAG) concentrations, inducing conventional protein kinase C (PKC θ) activation, and IRS 1/2 serine/threonine phosphorylation [49]. IRS serine phosphorylation reduces PI3K activation, an essential insulin sensitivity-related protein in skeletal muscle tissues [50].

Regarding the pancreatic islet insulin levels and β-cell sizes, it was observed], a greater immunoreactivity in the MIT group in comparison to C group (Fig. 5A) as was previously shown [3]. Treadmill exercise elevates AMPK phosphorylation in the rat pancreatic islets [35] and the AMPK activity, and inhibits insulin secretion in rodents [51], [35]. Although the mechanism by which BDNF-induced AMPK activation in the pancreatic islets is not completely clear, our results indicated a similar mechanism to that observed in skeletal muscle [29]. We could hypothesize an inhibition of the BDNF–AMPK axis to inhibit AMPK activity in MITK rats, leading to increased insulin secretion reflected by a reduced insulin islet immunostaining (Fig. 5A). Nevertheless, the reduced levels of immunostaining in the HIT and HITK groups indicated a possible reduction in insulin expression. In Wistar rats, treadmill running exercise was shown to increase Ca^2+^/calmodulin-dependent protein kinase (CaMKK) activity in an exercise-dependent manner [35], that inhibits insulin expression [52]. It is possibly that this protein activity could be increased in the HIT and HITK groups in comparison to the MIT, MITK, and C groups. Furthermore, the pancreatic islet areas in the MIT and HIT groups were bigger than those in C, MITK, and HITK groups (Fig. 5B). Increased insulin tolerance would reduce both, the demand for β-cell hormone secretion [53] and hormone synthesis [54], resulting in a smaller stress level and greater cell size among β-cells [55]. The lower levels of insulin tolerance observed in C, MITK, and HITK groups (Fig. 4B) probably induced greater levels of insulin synthesis/secretion and greater β-cell stress.

Importantly, we discarded that a TrkB inhibitor (K252a) alone affected the β-cell mass (Fig. 5A and B). The BDNF action *per se* did not increase insulin signaling in peripheral tissues [22], and it is known that exercise augments the insulin sensitivity in rodents [45], [46]. Physical exercise has positive effects on insulin signaling at β-cell level that results in a bigger β-cell mass in rodents [56], [57], [58], [55]. Taking into account these data, we suggested that in our exercised groups there is a synergistic action between BDNF and insulin to produce bigger β-cells and expanded β-cell mass (pancreatic islet size). In our trained groups with K252a, the BDNF insulin action was not enhanced to elevate β-cell mass.

Finally, our results did not exclude the participation of BDNF in islet cell apoptotic regulation as a potential cause of the smaller islet areas observed in MITK, HITK, and C groups, as reported in response to nervous growth factor activity [59]. Glycogen levels significantly increased after insulin administration in MIT and MITK groups when compared to C group (Fig. 6). This is likely due to increased insulin sensitivity induced by physical exercise in the MIT and MITK groups [9], [7], in which glycogen synthesis rose [60], [61]. Because glycogen levels were similar in MIT and MITK groups, it is unlikely that BDNF participates in glycogen increase as it occurs in diabetic rodents [22]. The lack of BDNF effect on hepatic glycogen could be explained by a lower TrkB expression in the liver, as happen in muscle after running [62]. In the HIT and HITK groups, the hepatic glycogen levels were similar to those found in rats from C group. During endurance training occurs a preponderant effect of glycogenolysis relative to glucogenesis [63]. Although we used a control group in all the experiments (GTT, ITT and liver glycogen), we cannot discard the possibility that the time interval between the different metabolic tests could have influenced in our results.

## Conclusion

To the best of our knowledge, the present study is the first to determine that chronic exercise could increase basal plasma BDNF levels in a healthy rat model. During chronic exercise, TrkB activity was necessary to regulate insulin tolerance and pancreatic islet size. In addition, our data demonstrated the presence of an exercise intensity threshold for BDNF expression in a slow skeletal muscle.
