# Correction: Chronic Exercise Increases Plasma Brain-Derived Neurotrophic Factor Levels, Pancreatic Islet Size, and Insulin Tolerance in a TrkB-Dependent Manner

**DOI:** 10.1371/journal.pone.0119047

**Published:** 2015-03-10

**Authors:** The PLOS ONE Staff

Fig. 5 is incorrect. The authors have provided a corrected version here.

**Fig 5:**
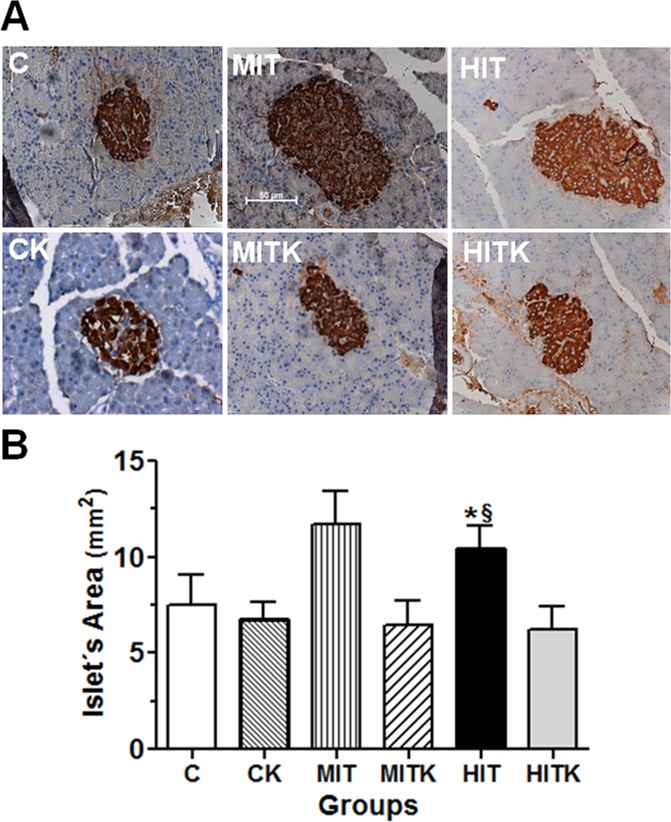
Pancreatic immunohistochemistry, effects of an 8-week treadmill exercise regimen in studied rats. (A) Insulin distribution and (B) pancreatic islet size. Light micrographs reveal the staining patterns of the pancreatic islets; the pancreatic islet areas (mm^2^) were evaluated in 209 islets. C, control sedentary group; CK sedentary rats that received K252a as in MITK and HITK groups; MIT, medium-intensity training rats; MITK, as MIT with a TrkB inhibitor (K252a) injection; HIT, high-intensity training rats; HITK, as HIT with a TrkB inhibitor injection; data are shown as means ± S.E. *p<0.05 *vs*. C; ^§^ *p*<0.05 *vs*.HITK; ANOVA and Tukey’ tests.
